# Bibliographical Notices

**Published:** 1842-04-01

**Authors:** 


### Periscope;

OR,

### CIRCUMSPECTIVE REVIEW.

" Ore trahit quodcunque potest, atque addit acervo."

### Notices of some New Works.

An Exposition of the Pathology of Hysteria, of Hysterical Amaurosis, &c. By *Ed. Oct. Hocken,* M.D. Duodecimo, pp. 32. Highley, Fleet Street, 1842.

Hysteria may fairly compete with Dyspepsia in the number of Proteian forms which it can assume—in the irregularity and caprice of its course—in the obstinacy of its nature and treatment. Our readers are aware that the substance of this Essay has already seen the light on both sides of the Tweed; and therefore we may be excused for not going into a very minute analysis of its contents—notwithstanding the seductive averment in the first page:—" The subject matter of the following Essay is perfectly original on my part, and derived solely from a close and unprejudiced observation of Nature." It is true that Dr. H. has recently met with something very much resembling his own doctrine, in one of those old musty authors, who occasionally disturb the mental complacency of modern discoverers. But he declares, and we fully credit the assertion, that he was totally ignorant of " Truka's Historea Amauroseos," at the time of his writing.

In Dr. Hocken's " definition " of hysteria, he seems to have taken especial care to avoid that horn of the dilemma on which so many literary travellers have been impaled.

——— dum *brevis* esse laboro,
*Obscurus* fio.———

" *Definition.*—We may define hysteria to consist of such a condition of the general nervous system, original or acquired, as renders it capable of simulating most local diseases, of complicating them in their progress, and modifying them in their usual phenomena; or the general nervous system itself may be the theatre of its operations, being then attended with general convulsions, and complicated with symptoms derived from the impeded performance of the functions of particular organs: the condition of the nervous system in its two extremes, varying from mere increased excitability and mobility to complete coma or catalepsy. Affections of an hysterical character are usually marked by great and sudden changes in the display of the mind's affections and sensibilities, by tears and laughter, by anger and joy, despair and hope; by a copious flow of limpid urine, nearly colourless, borborygmi and flatulence, the sense of a globular body rising from the abdomen into the throat (globus) with feelings of suffocation, and frequent efforts to swallow, and a circumscribed pain in the temple, severe in character, denominated clavus. The constitutional and local phenomena most frequently occur in females shortly after puberty, being rarer in the adult, rarely but still occasionally seen in the male.

Such I believe to be a sufficiently wide definition of hysteria."

We think so too. It is the longest " definition " we have ever met, and, in fact, it is a *description* of the malady in its more open and unequivocal character. Nor *do we* find fault with Dr. H.'s *lengthy* definition—except the vagueness of the first half, which might be omitted with very little loss. After all, the whole of our author's pamphlet—or at least nineteen-twentieths of it, is on the subject of hysterical amaurosis, with scarcely anything on its various other forms. This is, perhaps, all for the best.

"*Acute Hysterical Amaurosis.*—The amaurosis from hysteria may occur as an acute as well as a chronic affection; and under these circumstances we almost invariably find that derangements of the primæ viæ, of an acute or chronic character, have proved the immediate exciting causes, aided or called into action by mental excitement, slight bodily injury or fright. In these acute attacks we almost invariably meet with anomalous head symptoms, which precede and accompany the development and course of the amaurotic phenomena; and in a case which I saw from its commencement to its termination, many other local symptoms of a distinctly nervous character appeared and disappeared suddenly during its progress. This is one of the peculiarities of such cases, namely, that the symptoms come on with an intensity and hurry never marked in common inflammation; they alternate in character, disappear or are alleviated most rapidly from improvement in the condition of the intestinal canal, or the complete correction of the existing condition.

In the case to which I have just alluded, the amaurotic confusion and distress of vision came on in connexion with severe head symptoms, and a deranged condition of the abdominal viscera; this, with the other painful and deceptive phenomena readily yielded to remedies by which the alimentary canal was mildly but efficiently cleared out, the nervous system calmed, and the local abdominal irritation relieved."

An illustrative case is detailed, of which the following are the chief particulars:—

"At a period when the bowels were obstinately confined, about the seventh month of utero-gestation, she received a slight shock from tripping over the last stair in her descent, and was still further excited in the afternoon of the same day, by quarrelling with her husband. Under these circumstances she was attacked with a severe rigor, followed by heat of skin, a rapid, jerking, but soft and feeble pulse, thirst and dryness of the throat—there was the most violent cephalalgia of a pulsating kind, especially complained of over the brows; intolerance of light and amaurosis—the sight being imperfect, confused, and dazzling, and she was impatient of even the slightest noise; but the countenance was collapsed and pale, and she complained of sensations of syncope and vertigo on being raised into the erect posture; the abdomen was distended and slightly painful; the urine pale and abundant; the tongue moist but thickly coated, with a brownish slimy fur; the respiration accelerated, and the breath highly offensive."

A dose of five grains of calomel, with fifteen of rhubarb, and some pulvis aromaticus was given internally, while an anodyne terebinthinate enema was thrown up into the colon. A large quantity of highly offensive dark-coloured feculence was passed, containing scybalous masses, and with immediate relief. Several cases are detailed—some from the writings of others—some from the author's own observation.

The following is Dr. H.'s description of chronic hysterical amaurosis.

"The symptoms which characterise this form of amaurosis are the following, dividing them into objective and subjective:—Spasmodic contraction of the orbicular muscles of the eyelids, especially on exposure to bright light; evident intolerance of the influence of light on the retina—a condition which occasions a sense of most distressing anxiety, profuse lachrymation, and firm spasmodic closure of the eyelid when we attempt to make an examination of the organ; and if we succeed in raising the lid, which is readily effected if the eye has been quiet for some time before, we find the globe rotated upwards and outwards, still further to protect the suffering retina from light. The globe itself when exposed presents nothing unnatural: there is not the slightest trace of any zonular, pinkish, hair-like arrangement of vessels around the cornea, which run parallel to within a short distance of its circumference, no conjunctival vascularity when the lid is first raised—but if the exposure be continued for any length of time in a strong light, then scarlet redness comes on, depending on the irritation and consequent hyperæmia of the conjunctival vessels—but this quickly disappears on the removal of the exciting cause. The pupils (for the affection always exists in both eyes at the same time) are contracted, and this in proportion to the degree of light present, and the degree of morbid sensibility in the retina; but they contract and dilate fully and freely in either eye by alternation of light and shade—proving that the state of the iris is dependent merely on its connexion with the condition of the retina, and not on any specific affection of its own motile powers. The texture of the iris retains its healthy brilliancy and radiated appearance, its normal structure and colour, nor is there any irregularity in the pupillary edge, no retraction with bulging of the iris forwards, nor any alteration in the situation of the pupil. The vitreous humour retains its healthy character: hence the eye-balls are neither flaccid nor hard and prominent; there is no greenish discolouration, no deep-seated opacity. The lens, the cornea, the aqueous capsule, and the aqueous humour are all healthy; and thus a local examination would lead to no knowledge of the pathological causation of the other symptoms which we learn from the patient—the eye and its appendages appear perfectly normal in their structure, but the retina and conjunctiva, and sympathetically the lachrymal gland and orbicular muscle morbidly irritable and sensitive.

The patient, when questioned about her local symptoms, complains of defective vision and great sensibility to the influence of light; these being invariably connected, so that it is impossible to separate them, and specify how much the one is dependent on the presence of the other: thus when she (for I have never seen the affection clearly defined in males) attempts to view any obiect, it is at first imperfectly defined and clothed in mist; but the increased sensibility of the retina, the lachrymation and the spasmodic closure of the lids soon obstruct vision completely. In the dark the intolerance of light diminishes, but never disappears entirely, and the sight still continues imperfect, but is nevertheless improved: but, on the contrary, in bright sunshine, or in an intensely illuminated apartment, she sees little or nothing, and strenuously closes the lids like a child suffering from strumous photophobia and blepharospasmus. The appearance of luminous or dark spectra is unusual, except occasionally from looking at white bodies, or those highly reflective of light, or on first entering a brightly lighted apartment; but they never trouble the patient as in retinitis, nor is the eye-ball itself the seat of any uneasiness or pain."

With the above, the general and local symptoms of hysteria are combined.

In respect to the pathology of hysteria, the following contains the pith of all we can find in the pamphlet.

"*Pathology.*—I may enquire what relation those local derangements termed 'local hysteria' bear to that general affection of the nervous system, to which the specific title of 'hysteria' is applied? Are we to regard them as strictly local derangements of innervation, produced, but modified, by the same causes which induce the hysteria itself (if any local source of irritation be present), or symptomatic of that general condition of the nervous system which constitutes hysteria? or lastly a reflected action from derangement of the nervous centres? My own opinion is, that the amaurosis is a strictly local hysterical affection, analogous to those local nervous disturbances produced in the integuments, &c. covering the spine, and in many other situations—similating disease of the part or joint, which are primarily dependent on the hysteria for their origin and existence, but may become localised and independent,—just in the same manner as the local affections in acute rheumatism are primarily but local manifestations of a general disease, yet by continuance become independent of their constitutional origin, and may even go on to produce the disorganizing results of common inflammation, which the local affections never produce whilst they retain their dependent and specific character: hence in those cases where symptoms denote the amaurosis as specific, the local irritation (should there be any) whether it be abdominal or uterine, bears no other relation to it than as an excitant of the hysterical derangement of the general nervous system, on which the local depends. If these propositions be regarded as satisfactory, they will bear me out in stating, before I proceed with the subject of diagnosis, and to the elucidation of Mr. Middlemore's queries,—1st. That the amaurosis should be attributed to the hysteria, and reckoned among those local anomalous derangements which the protean disorder is so fond of originating: 2nd. In reply to the enquiry—what share do abdominal or uterine derangements take in the production of the local and general symptoms?—we must reply that we cannot trace them both to the local irritation, or attribute them both to one and the same cause, since the hysteria is not necessarily connected with any local source of irritation, and certainly the amaurosis is not one of a simply sympathetic origin."

As far, then, as the general pathology of HYSTERIA is concerned, we cannot say that our author has given us much more insight into the matter than we possessed before we perused the pamphlet. Its simulation of amaurosis is more extended and delineated than in any notice or monograph on the subject, that we have seen—and this is the chief merit of the pamphlet.

Perhaps we might just be allowed to hint to Dr. Hocken, and very many others, that many things are long known before they appear in print—and that the first who publishes is not always the first who discovers or invents. We can safely say that, for very many years back, the fact of hysteria occasionally simulating amaurosis, was familiar to us and hundreds of other practitioners. No man knows this better than Sir Benjamin Brodie himself, to whom Dr. H. has dedicated his monograph. At the same time, we are quite ready to grant that Dr. H. is entitled to the thanks of the profession for collecting his scattered thoughts and papers into a focus, and thus bringing before the public some important facts for their consideration and reflection.

---

### A BRIEF MEMOIR OF GEORGE BIRKBECK, M.D. By *Henry Clutterbuck*, M.D. Highley, 1s. 1842.

IT is not to the "storied urns" of the great—the rude sepulchral stones of the humble—

"With uncouth rhymes and shapeless sculpture deck'd,"

—the "*eloges*" pronounced over the biers of departed genius—nor to the "brief memoirs" of the dead, penned by friendship, poured forth by gratitude, perhaps prompted by pride—that we are to look for a true and just portraiture of those who have dropped from amongst us into the great ocean of eternity! The winding-sheet possesses the almost miraculous power of shrouding our failings and revealing our merits. No sooner does the vital spark forsake its tenement of clay, than our enemies cease their vituperations, or even join in our praise; while many of our dearest friends begin to recollect a thousand good actions, or amiable traits of character which had slipped their memory, or, at all events, never escaped their lips, till we are insensible alike to censure or flattery!

The subject of the present Memoir was one of those few who have no enemies, even where they have opponents in this world. Every one knows what a strenuous advocate Dr. Birkbeck was for the diffusion of knowledge among the middling and lower classes of society—especially among those who were formerly called mechanics, but who are now designated as operatives. There is a very large and influential class in this—perhaps in all countries—who look upon this diffusion of knowledge as extremely dangerous, and who, by every means in their power—more frequently by concealed than by overt acts—counteract the spread of learning beyond the pale of those who do *not* work for their bread by manual labour. Dr. Birkbeck had, therefore, many opponents among the aristocracy—but, we believe, no enemies. We well recollect the time when one of the greatest wits of the day, but whose wit will never more " set the table in a roar,"* while ridiculing the idea of teaching mechanics the elements and practical application of the sciences, could find no other weapons than those of nicknames, to assail the popular lecturer, whom he denominated, " Dr. BRICKBAT, of the Mechanics' Institution."

We were well acquainted with our deceased confrere for more than twenty years, and can confirm the sentiments which Dr. Clutterbuck, so long his coadjutor at the Aldersgate Dispensary, has expressed towards him.

" In his *professional* character, Dr. Birkbeck's claim to notice was of the highest stamp. Of this, I consider myself entitled to judge in some degree, from my almost daily communication with him for a long series of years. Acute in observation, discriminating in judgment, patient and cautious in prescribing and administering remedies, he was, as might be expected, eminently successful in practice; thereby, as well as by suavity of demeanour, ensuring the entire confidence of his patients. He was thoroughly imbued with a knowledge of the *principles* and *practice* of our art, as at present subsisting; and, had his time and talents been directed *exclusively*, or even *principally*, to the study of medicine, he would unquestionably have enlarged the boundaries of the science; and thus have contributed to its extrication from some part at least of the obscurity, in which it is at present but too deeply involved.

As a man, Dr. Birkbeck was simple, unassuming, and artless in his manners; of unbounded benevolence, and inflexible integrity. He was beloved, as well as esteemed, by a large circle of private friends—admired, respected, and lamented, by multitudes of all ranks, who had profited by his instruction, or by his benevolence;—and, I may add, he was almost adored in his domestic circle."

Though abstemious, even to an extreme, Dr. Birkbeck was destined to experience severe sufferings for some time before his death. For a long time he laboured under chronic hepatitis, and it is hinted by the talented Biographer that the disease was rather aggravated than relieved by the remedies prescribed by the patient's friends. He recovered under more rational treatment, but ultimately died of disease of the bladder, with enlargement of the prostate gland.

Although Dr. Birkbeck's eminent abilities, coupled with the great popularity which he acquired among the operatives, were calculated to lead him into extensive practice, yet, that practice was not so wide as might have been expected. The cause, however, is well known to those who have a knowledge of the world, as it respects the medical profession. All public reputation for extra medical studies, and reputed devotion to them, are sure to injure a physician in the practice of medicine. Even those studies which are auxiliary, and closely allied to physic, as chemistry, botany, &c. are far from calculated to spread the practice of his profession, if the public get an idea that they engross much of a medical man's attention. The world conclude (and perhaps not very erroneously) that the practice—the mere observation of diseases, and application of remedies—is enough for even a long life and the most assiduous attention. They conclude as a corollary, that addiction to any study, not essentially connected with practical medicine, has a tendency to absorb too much of the individual's time and thought, and wean zeal from the " one thing needful." It is

* Theodore Hook, then Editor of the " JOHN BULL."

probably from this notion, however mistaken, that we see the public consulting medical men, who have long declined in their mental faculties, and still longer ceased to keep pace with the progress of medical science, in preference to those who are in the vigour of their intellect, and who know every thing, true or false, that has transpired in the world around them.

In conclusion, we may state, that this sensible and modest Memoir on the life of a departed friend, does equal honour to the head and heart of its talented author, Dr. Clutterbuck.

---

A TREATISE ON DISLOCATIONS AND FRACTURES OF THE JOINTS. By Sir *Astley Cooper*, Bart. F.R.S. Serjeant-Surgeon to the King, &c. Edited by *Bransby B. Cooper*, F.R.S. Surgeon to Guy's Hospital. London, Churchill, pp. 576. 1842.

The present will be found a much more convenient edition of this invaluable work than its predecessors. It is in the octavo form instead of the quarto, and not only is more portable, but more cheap. Although new matter and new illustrations have been added, the price has been reduced from two guineas to twenty shillings, a circumstance which, in these hard times, will be unanimously voted an improvement.

The editor, Mr. Bransby Cooper, observes that, from papers (communicated to him), as well as from his own practice, several additional cases have been selected for the present edition; and much new matter has been added, which was derived from Sir Astley Cooper himself.

In order to admit of these additions, and at the same time to preserve the matter within the limits of the present volume, it has been thought necessary in some few instances to condense the original. It is, however, proper to mention, that the additional cases which were detailed in the preface to the last edition are now introduced into the body of the work, under the appropriate heads to which they refer.

Mr. Bagg, who may be styled the student's friend, has been invited to contribute his clever pencil to the illustration of the work. And he has done so with effect. The Editor informs us, that the reader will find the delineations copied from the quarto edition to be even more graphic and perspicuous than the originals; while the illustrations now for the first time introduced into the work are equally correct, clear and expressive. The advantages of such engravings being placed in immediate connexion with the portion of text which they are intended to elucidate, will not pass unnoticed by those who have felt the inconvenience of having to search at the end of the volume for each plate to which reference occurs in the text.

After the fiat of the profession, it would be absurd in us to eulogise Sir Astley Cooper's work on Fractures and Dislocations.

It is a national one, and will probably subsist as long as English surgery. But still additions to it may unquestionably be made, and if corrections are not needed emendations may be useful. The additions which have been ventured on by Mr. Cooper seem to us, so far as we have observed them, to be judicious and useful. Perhaps a little more variety might have been displayed in reference to the means of surgical treatment, but this is a good fault. A lengthy catalogue of names and methods more frequently embarrasses the student than assists him, and one good and practical direction is worth a dozen doubtful ones.

Altogether, Mr. Cooper deserves well for this edition, and we are convinced it will be popular.

